# Influence of Different pH Values on Gels Produced from Tea Polyphenols and Low Acyl Gellan Gum

**DOI:** 10.3390/gels9050368

**Published:** 2023-04-28

**Authors:** Fangyan Zhang, Xiangcun Wang, Na Guo, Huanhuan Dai, Yimei Wang, Yiwei Sun, Guilan Zhu

**Affiliations:** 1 Department of Biological and Food Engineering, Hefei Normal University, Lian Hua Road, Hefei 230601, China; 2 Anhui Engineering Laboratory for Medicinal and Food Homologous Natural Resoures Exploration, Hefei 230601, China

**Keywords:** tea polyphenols, low acyl gellan gum, pH

## Abstract

To explore the influence of pH values on the properties of a compound system containing tea polyphenols (TPs) and low acyl gellan gum (LGG), the color, texture characteristics, rheological properties, water holding capacity (WHC), and microstructure of the compound system were measured. The results showed that the pH value noticeably affects the color and WHC of compound gels. Gels from pH 3 to 5 were yellow, gels from pH 6 to 7 were light brown, and gels from pH 8 to 9 were dark brown. The hardness decreased and the springiness increased with an increase in pH. The steady shear results showed that the viscosity of the compound gel solutions with different pH values decreased with increasing shear rates, indicating that all of the compound gel solutions were pseudoplastic fluids. The dynamic frequency results showed that the *G*′ and *G*″ of the compound gel solutions gradually decreased with increasing pH and that *G*′ was higher than *G*″. No phase transition occurred in the gel state under heating or cooling conditions at pH 3, indicating that the pH 3 compound gel solution was elastic. The WHC of the pH 3 compound gel was only 79.97% but the WHC of compound gels pH 6 and pH 7 was almost 100%. The network structure of the gels was dense and stable under acidic conditions. The electrostatic repulsion between the carboxyl groups was shielded by H^+^ with increasing acidity. The three-dimensional network structure was easily formed by an increase in the interactions of the hydrogen bonds.

## 1. Introduction

Tea polyphenols (TPs) are natural antioxidants that are the main active ingredients in tea. Considering their good antioxidant, antiaging, and anticancer [1,2,3] functions, TPs have been widely used as a therapeutic agent or food supplement. However, their poor absorption, easy decomposition, and low metabolic transformation under physiological conditions limit their active functions [4]. TPs are sensitive to light, temperature, and pH, which makes them very unstable and easily oxidized [5,6,7]. To increase the effective use of TPs, several drawbacks must be overcome, such as oxidation and instability. Polyphenols and polysaccharides can form complexes through intermolecular interactions. In addition, the combination of TPs with hydrophilic polysaccharides can effectively prevent the oxidation process of TPs and improve their bioavailability [8].

**Figure 1:**
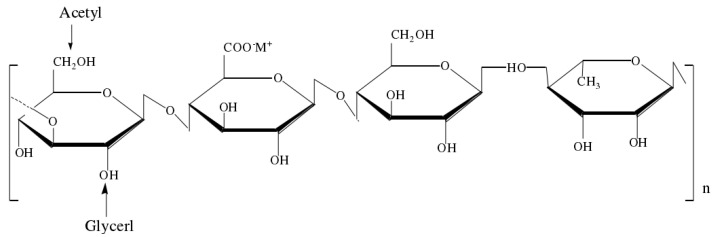
Structure of gellan gum.

Gellan gum (GG) is an anionic linear exopolysaccharide produced by the fermentation of *Sphingomonas paucimobilis*. It is a widely used microbial polysaccharide after xanthan gum. Its main chain is composed of 2 molecules of D-glucose, 1 molecule of D-glucuronic acid, and 1 molecule of L-rhamnose [9] in a 2:1:1 ratio linked together to form the linear primary structure [D-Glc(β1→4)D-GlcA(β1→4)D-Glc(β1→4)L-Rha(α1→3)]n [10]. The structure of GG is shown in Figure 1.

Compared with plant gels, such as agar, gelatin, and pectin, and microbial gels, such as xanthan gum and pullulan, GG has certain advantages such as easy gel formation, high colloidal transparency, and resistance to enzymatic hydrolysis. It is mainly used as a gelling agent, stabilizer, suspending agent, and film-forming agent [11,12]. GG is mainly classified as a high acyl GG (HGG) or low acyl GG (LGG) and LGG is more widely used in the food industry [13,14]. Because the molecular structure of HGG is rich in acyl groups, it can form an elastic and tough opaque gel, while LGG can form a harder and more brittle transparent gel [15]. However, LGG is more susceptible to pH than HGG [16]. Therefore, our experiment selected LGG as our study material. TPs are rich in phenolic hydroxyl groups and can form hydrogen bonds with GG molecules to improve the viscosity and elasticity of the gel compound. The rich carboxyl and acyl groups in the molecular structure of GG also have a good compounding effect with TPs, which can delay the oxidation process of TPs and play a role in stabilization and slow release [17].

pH mainly affects the charge of biomacromolecules in gel molecules and the interactions between polymers. The binding ability of TPs to oat-β-glucan was the strongest at pH 6.0 [18]. pH was the most important factor that affected the interaction between cellulose and cyanidin-3-glucoside, and the binding ability of cyanidin-3-glucoside and cellulose varies with pH (3.0–5.0); the binding capacity of the two decreased when the pH increased to 7.0, which may be caused by the chemical structure of anthocyanins changing with pH [19]. pH (2.0–4.5) can also significantly affect the interaction between anthocyanins and pectics; the binding effect of the two is the strongest at pH 3.6 and the affinity between the two is relatively weak at other pH values [20]. Therefore, pH affects the properties of the compound gel formed by TPs and LGG.

In order to explore the effect of different pH values on the gel properties of the compound gels formed by TPs and LGG, we tested the color, texture characteristics, rheological properties, WHC, and microstructure of the compound gels with TPA, SEM, and FTIR. The results will provide a theoretical basis for the application of compound gels produced by TPs and LGG in real food systems and effectively improve the nutritional and functional properties of food.

## 2. Results and Discussion

### 2.1. Surface Color Analysis

**Figure 2:**
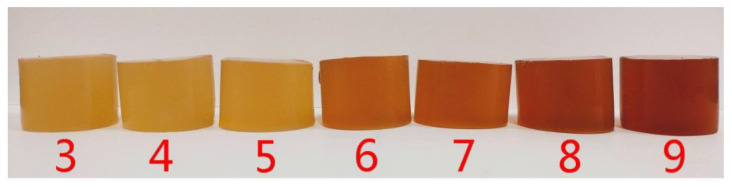
Color change of TP-LGG compound gels with different pH values. (number 3–9 mean pH values).

We first examined the influence of pH on the color of compound gels because color measurements could enable us to distinguish the effect of pH on the TP-LGG compound gels. The color changes of the compound gels under different pH conditions are shown in Table 1 and Figure 2.

Table 1 shows that the L*, a*, and Δ*E* values increased, whereas the b* values decreased with increased pH. The higher the L* value is, the lighter the gel is [21,22]. The indicators L*, a*, b*, and Δ*E* were positive values, indicating that the compound gels gradually changed from yellow to dark brown with increased pH. This was consistent with the observations in Figure 2. TPs were relatively stable under acidic conditions whereas they were very unstable in neutral and alkaline solutions and decomposed in a few minutes [6], which caused the color of the compound gels to darken.

In the pH range of 3–5, the b* value of the samples changed, and the color of the compound gels was yellow. However, when the pH was over 6, the b* value decreased with increased pH. The compound gels became darker, considering that TPs are easily oxidized under alkaline conditions. At the same time, the difference in Δ*E* approached 3, indicating that the change in pH resulted in a visible change in the color of the compound gels.

The LGG gel was colorless and transparent. However, when TPs were added to LGG, the compound gels became yellow because of the presence of flavonoids and anthocyanins in the TPs. The natural pH of the TP-LGG gel was 5.2, and the compound gel was pale yellow. In the pH range of 3–5, the compound gels were still yellow. At pH 6–7, the compound gels were light brown. At pH 8–9, the compound gels were dark brown. This color change was expected because with the a* value increased and the b* value decreased, the red was deepened and the yellow was weakened so that the color of the compound gels changed as shown in Figure 2. TP solutions are oxidized into quinones mainly through oxidation and dimer formation, which deepened the color of the compound gels. The TP solution degraded irreversibly to a yellowish-brown solution due to the deterioration products, and this result was consistent with previous findings [23].

### 2.2. TPA Results

**Figure 3:**
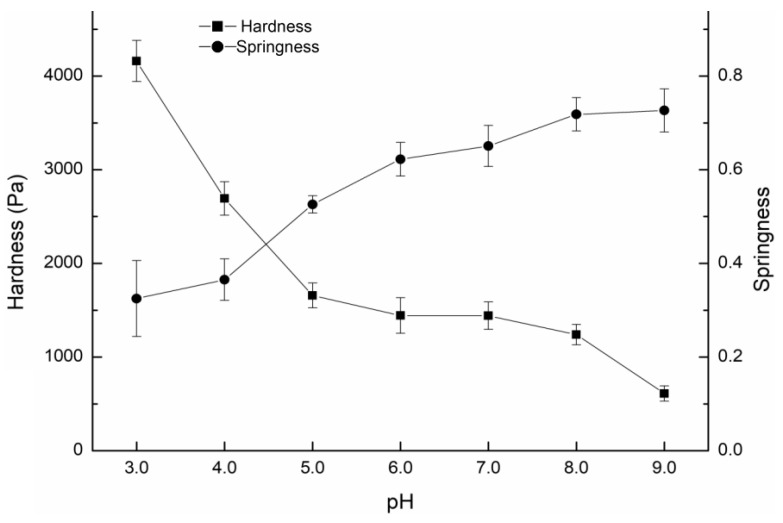
Hardness and springiness of the compound gels with different pH values.

According to Figure 3, both the hardness and springiness of the compound gels with different pH values were significantly affected. The hardness decreased and the springiness increased as the pH rose from 3 to 9. The hardness of the compound gel (pH 3) reached a maximum value of 4216.377 g. Then, as the pH increased to 6, the hardness decreased abruptly. When the pH rose from 6 to 9, the hardness decreased slowly. This was consistent with previous findings that reported that hydrochloric acid acidifies LGG to make acid-sensitive colloids and found that the hardness and strength of the gel increased significantly when the pH decreased from 5 to 3 [24]. As the acidity of the compound gels continued to decrease, the concentration of H^+^ in the system continued to increase. This shielded the negatively charged carboxyl groups in the molecular structure of GG, reduced the mutual repulsion between polymers, and facilitated the formation of a three-dimensional network structure, thus enhancing the hardness of the compounded gel. The springiness of the gel was stable in the pH range of 6–9. However, the springiness of the compound gel was greatly weakened, and it became hard and brittle with the decrease in pH from 6 to 3, and reached a minimum at pH 3. Under weakly acidic conditions, the gel formed a dense three-dimensional network structure, making the gel more resistant to external impact. However, when the structure was completely destroyed, the network structure was more difficult to restore, making the gel less elastic.

### 2.3. Rheological Analyses

#### 2.3.1. Steady Shear Analyses

**Figure 4:**
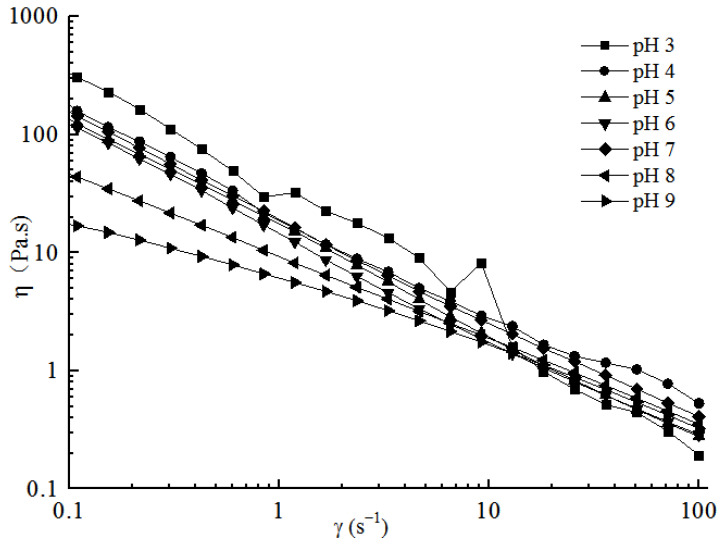
**Figure 4.** Variation in the viscosity of the gel solutions with different pH values.

During the stirring shear process, the molecular chains of TPLGG were straightened or dispersed under external forces, leading to an increase in the fluidity of the systems and a decrease in viscosity. This phenomenon is shear thinning and is commonly used to indicate the shear stability of systems during the shear process. The viscosity and shear stress of the TP-LGG compound systems is shown in Figure 4. As shown in Figure 4, the systems exhibited high viscosity at low shear rates, followed by a linear decrease in viscosity with the increasing shear rate, exhibiting a typical shear thinning behavior and indicating that the TP-LGG compound gel solutions were non-Newtonian fluids. The shear thinning behavior between TPs and LGG was mainly attributed to the destruction of intramolecular or intermolecular interactions [25,26] which caused a decrease in intermolecular electrostatic repulsion and an increase in mutual aggregation. At the same shear rate, the viscosity decreased as the pH increased from 3 to 9, and the viscosity of the compound gel solution was the highest at pH 3. H^+^ could enhance the electrostatic repulsion between the negatively charged carboxyl groups in the LGG molecule under strongly acidic conditions, which made it easier to form a three-dimensional network structure in the gel solution. Strong interaction forces, such as hydrogen bonds between molecules, increased the viscosity of the compound gel solutions. The result was similar to the result of Picone and Cunha [27] who found that the viscosity of the gel increased when the pH of the LGG system was reduced from 5.3 to 3.5.

#### 2.3.2. Dynamic Frequency Sweep Analyses

**Figure 5:**
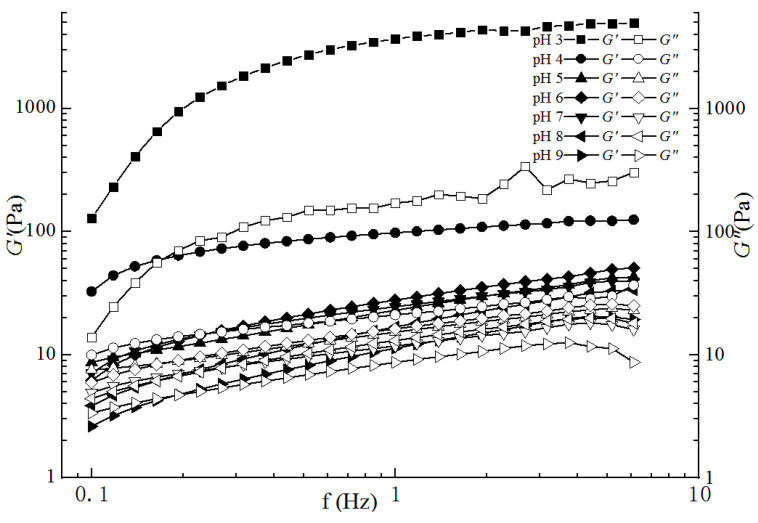
Frequency dependence of the compound gel solutions with different pH values.

As shown in Figure 5, the viscoelastic *G*′ and *G*″ values of the compound gel solutions increased as the frequency rose from 0.01 to 6.105 Hz. The values of *G*′ and *G*″ decreased with the increase in pH from 3 to 9. In the range of pH 3–7, *G*′ was clearly higher than *G*″, especially in the gel solutions of pH 3 and pH 4. The compound gel solutions were obviously predominantly elastic. The high concentration of H^+^ in the system was conducive to the formation of a three-dimensional network structure, thereby enhancing the viscosity of the compound gel solutions. The *G*′ and *G*″ of the compound gel solutions at pH 8 and pH 9 were raised but the *G*″ were higher than *G*′ with low frequencies, and then *G*′ were still higher than *G*″ later. Therefore, the compound gel solutions mainly exhibited solid-like characteristics from pH 3 to 9. Chen et al. [28] also found that LGG exhibited solid properties under acidic conditions, which was consistent with the results of this experiment.

#### 2.3.3. Dynamic Temperature Scanning

**Figure 6:**
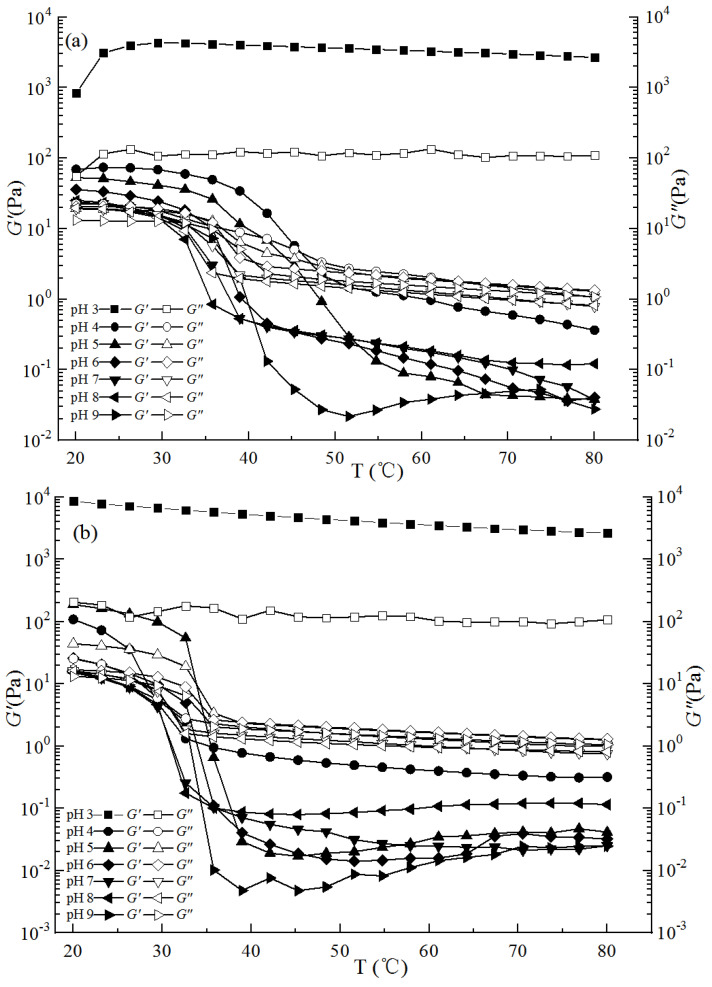
Effects of heating and cooling frequency on the *G*′ and *G*″ of the compound gel solutions with different pH values ((**a**) indicates the heating process; (**b**) indicates the cooling process).

As shown in Figure 6, whether in the process of heating or cooling, the *G*′ and *G*″ of all samples generally decreased with the increase in pH value, and the *G*′ and *G*″ of the pH 3 gel solution were the highest. During the heating process, except for the pH 3 gel solution, the *G*′ and *G*″ values of the other compound gels decreased and underwent a solid-to-liquid transition. As shown in Figure 6a, the increase in pH value reduced the melting temperature of the gels. During the cooling process, the *G*′ and *G*″ values of all the compound gels increased, and some gels underwent a liquid-to-solid transition. As shown in Figure 6b, the pH value has an effect on the freezing point of the compound gel during the cooling process. In comparison with the dynamic temperature scan curves under the two conditions, the gelling temperature under cooling conditions was lower than the melting temperature under heating conditions, and an obvious hysteresis phenomenon occurred. The compound gel solution at pH 3 had no phase transition and occurred in the gel state under both heating and cooling conditions, indicating that the pH 3 compound gel solution was elastic. Similar to the findings of Li et al. [11], who used glucono-δ-lactone to prepare thermally irreversible gels of LGG, decreasing pH is usually more effective in promoting gellan gelation.

### 2.4. WHC

**Figure 7:**
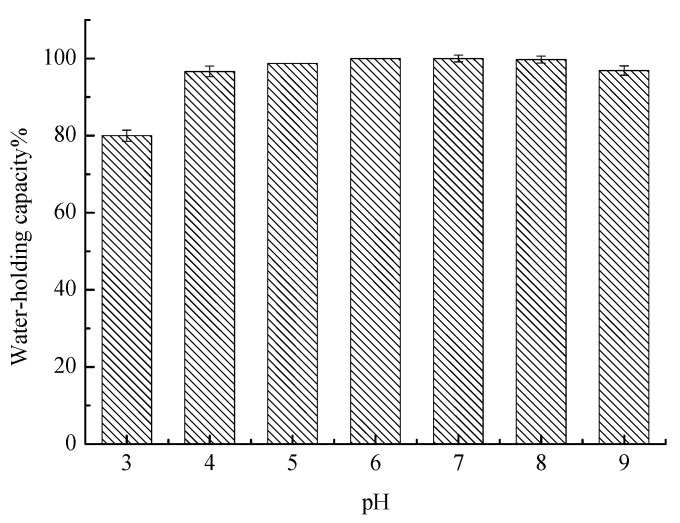
Effect of pH on the WHC of the compound gels.

The WHC of the compound gels with different pH values is shown in Figure 7. The WHC of the compound gels increased from pH values 3 to 6 and reached a maximum at pH 6. Interestingly, the WHC of the pH 6 compound gel was the same as that of the pH 7 compound gel, which is almost 100%. Then, the WHC of the compound gels began to decrease as the pH value increased from 7 to 9. The results showed that the WHC of the compound gels was relatively stable at pH 6–8. When the acidity was reduced to pH 5, the WHC of the gels started to decrease and was the lowest at pH 3, only 79.97%. The dissociation degree of the carboxyl group of the hydrophilic group in LGG decreased under acidic conditions. Consequently, the WHC of the gel decreased. The decrease in the WHC of the pH 9 compound gel can be attributed to the decreased hydrophilic groups such as hydroxyl groups caused by the oxidation of TPs, thus reducing the WHC of the compound gels. Previous studies suggested that the WHC was significantly reduced under the acidic condition of LGG [28].

### 2.5. Microstructure Analysis

**Figure 8:**
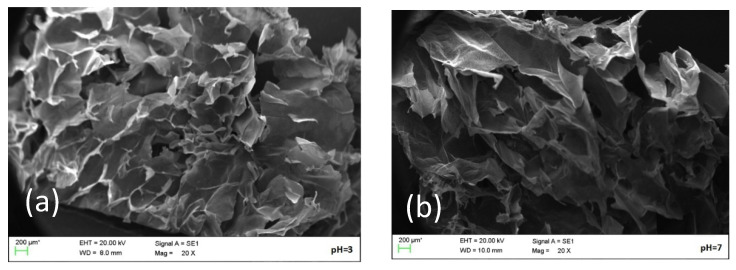

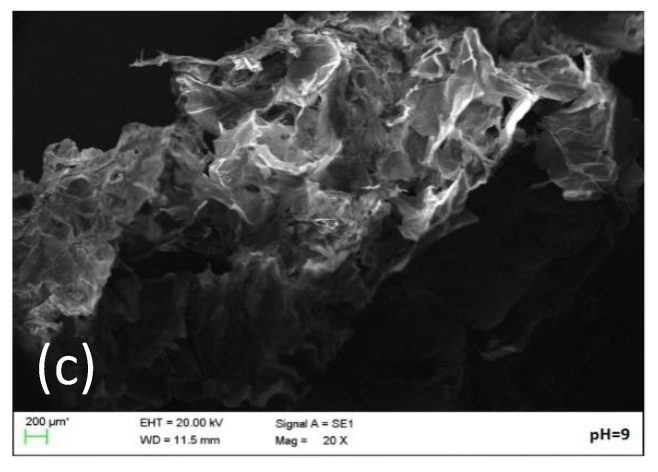
SEM images of compound gels with different pH values. ((**a**) means the compound gel with pH 3; (**b**) means the compound gel with pH 7; (**c**) means the compound gel with pH 9).

The SEM images revealed that pH affected the morphology of the compound gel system as seen in Figure 8. The microstructure of the compound gel at pH 3 (Figure 8a) was heterogeneous and porous, with numerous “water channels” inside the gel structure. H^+^ reduced the degree of dissociation of carboxyl groups and shielded the electrostatic repulsion between the carboxyl groups to promote the formation of a double helix structure which was conducive to the formation of a compact three-dimensional network structure [28]. Therefore, the WHC of the pH 3 gel was the weakest. However, the gel microstructure became more inhomogeneous as the pH increased. When the pH was 7 (Figure 8b), the internal structure of the compound gel was relatively stable, improving the WHC of the gel. At pH 9 (Figure 8c), the network structure formed in the internal structure of the compound gel was relatively disordered. Considering that the electrostatic repulsion between the carboxyl groups in LGG molecules was strong under alkaline conditions and that the double helix between polymers was degraded, the formation of a network structure was not induced. Meanwhile, TPs were oxidized and their interaction with LGG molecules was affected, resulting in the chaotic internal structure of the compound gels. The network structure of the LGG was weak under alkaline conditions according to electron microscope scanning [29]. Microscopy images showed that the pH had obvious effects on the microstructure of the compound gels.

### 2.6. FTIR Spectra Analysis

**Figure 9:**
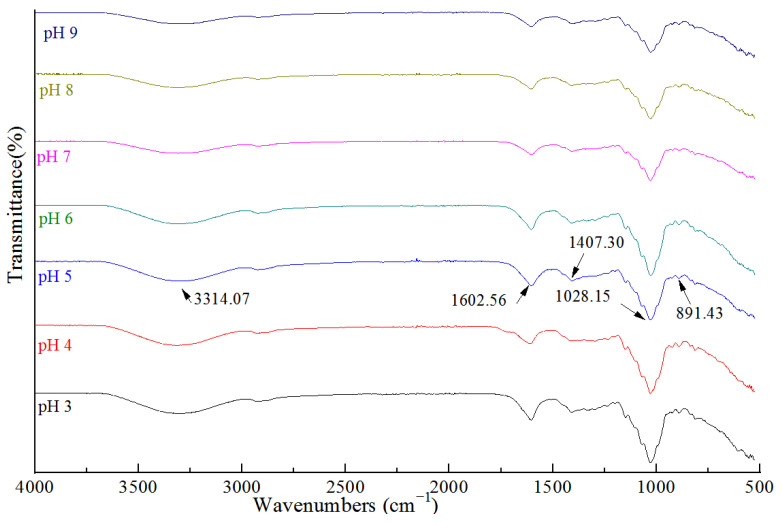
Fourier transform infrared spectra of the compound gels.

FTIR analysis was performed to confirm the cross-linking interactions between TPs and LGG. The infrared absorption spectra of the compound gels with different pH values are shown in Figure 9. The compound gels with different pH values had similar absorption peak shapes with slightly different intensities and positions. Although the sample was freeze-dried and still contained bound water, the wide absorption peak in the range of 3500–3100 cm^−1^ was mainly the superposition of the O–H stretching vibration and water in the gels. The stretching vibration of O–H near 3314.07 included inter-and intra-molecular interactions [29]. The hydroxyl groups of samples existed in an associative manner so the absorption peak was very wide. Under acidic conditions, more hydrogen bonds were generated between water molecules and between them and other components, resulting in an increase in the intensity of the O–H absorption peak [30]. A strong absorption near 1602.56 cm^−1^ could be attributed to the C–O stretching vibrations in the LGG molecule. A weak absorption near 1411.16 cm^−1^ could be attributed to the O-H bending vibration. The absorption peak near 1028.15 cm^−1^ was associated with the stretching vibration of C–O–C. The spectra of compound gels showed characteristic peaks near 1602.56 cm^−1^ (C–O stretching vibrations), 1407.30 cm^−1^ (O–H bending vibration), and 1021 cm^−1^ (C–O–C stretching) [31]. Overall, the intensity of the absorption peak weakened and slightly moved towards the low-frequency direction as the pH value increased, indicating that with the increase in H^+^ concentration, the dissociation degree of the carboxyl group was inhibited, and the electrostatic interaction between polymers decreased. Therefore, we speculated that the change in pH could not generate a new chemical bond between TPs and LGG. Based on the FTIR results, we concluded that TPs were compatible with LGG during the mixing and gelation stage due to intermolecular synergistic effects and hydrogen bonding [32].

## 3. Conclusions

The results of this work showed that pH affected the properties of TP-LGG compound gels. The compound gels turned from yellow to brown and then to dark brown with increasing pH. In addition, the hardness of the compound gels rose, and the springiness decreased continuously with increasing pH. The steady shear results showed that the viscosity of the compound gel solutions with different pH values decreased with increasing shear rates, indicating that the compound gel solutions were pseudoplastic fluids. The compound gel solutions mainly exhibited solid-like characteristics in the acidic and neutral ranges and exhibited liquid-like properties under weakly alkaline conditions. The compound gels also underwent a solid-to-liquid transition in the process of heating and cooling. The values of *G*′ and *G*″ decreased with increasing pH. The WHC of the gels was relatively stable and strong in the neutral and weakly alkaline ranges, but the WHC under acidic conditions was the weakest. Furthermore, the network structure of the compound gels was relatively dense and stable under acidic conditions and, finally, there were cross-linking reactions between TPs and LGG with increasing acidity.

## 4. Materials and Methods

### 4.1. Materials

LGG (food grade) was obtained from the Ruifeng (Henan, China). TPs (food grade) were obtained from the Naman (Nanjing, China). Hydrochloric acid and sodium hydroxide were obtained from Meifeng (Hefei, China).

### 4.2. Preparation of TP-LGG Compound Gels

A 1.0% (*w*/*w*) LGG solution and 0.15% (*w*/*w*) TP solution were prepared by dissolving them in deionized water and stirring at 90 °C to ensure complete powder hydration and dissolution. The pH of the system was adjusted to 3, 4, 5, 6, 7, 8, and 9 with 1.0 M NaOH or 1.0 M HCl solution. A pH meter (FE20K, Mettler Toledo, Leicester, UK) was used to measure the pH of the solution. Then, 0.15 mL of 2 M Ca^2+^ solution was added to the compound gel. Finally, the compound gel was placed in a plastic casing while it was still hot and stored in a refrigerator at 4 °C for 24 h after cooling.

### 4.3. Color Measurements

The color was measured as previously described [33,34], with some modifications. A colorimeter (CR-400, Konica Minolta, Tokyo, Japan) was used to observe the color changes of the gels. The color was denoted by the *a*, *b,* and *L* values, indicating redness/greenness, yellowness/blueness, and lightness, respectively. The machine was calibrated using a standard white tile, and the L*, a*, and b* values were obtained. The total color difference (Δ*E*) was calculated using the following equation:

$$\Delta E=\sqrt{{\left(\Delta L^{*}\right)}^{2}+{\left(\Delta a^{*}\right)}^{2}+{\left(\Delta b^{*}\right)}^{2}}$$

### 4.4. Texture Profile Analysis (TPA) of TP-LGG Compound Gels

The gels were cut into cylinders with a height of 25 mm and radius of 25 mm, and the TA-XT plus (Stable Micro Systems, Surry, UK) physical property analyzer was used for measurements. The parameters of the tests were set as follows: measure type, TPA; probe type, P/50; compression strain, 50%; pretest speed, 5.0 mm/s; test speed, 1.0 mm/s; and post-test speed, 5.0 mm/s. Based on the characteristics of the samples, data on hardness (g) and springiness (g) were recorded and used as the analysis index [25].

### 4.5. Rheological Measurements of TP-LGG Compound Gels

#### 4.5.1. Steady Shear Analyses

The steady rheology was tested as previously described [35,36], with some modifications. The rheological properties of the compound gel solutions were studied using a rheometer (HAAKE RS6000) with flat rotor model P35 TiL (diameter, 35 mm; gap, 1 mm). Samples were tested at 25 °C with steady shear rates in the range of 0.1–100 s^−1^, and the viscosity of the compound gel solutions was recorded.

#### 4.5.2. Dynamic Frequency Sweep Rheological Measurements

Frequency sweep measurements at a fixed amplitude of shear stress were carried out in the frequency range of 0.01–10 Hz at 25 ± 0.1 °C. The values of the storage modulus (*G*′) and loss modulus (*G*″) were recorded [36].

#### 4.5.3. Dynamic Temperature Sweep Rheological Measurements

Temperature sweeps were conducted at a heating rate of 5 °C/min, reduced from 80 °C to 20 °C, and finally increased from 20 °C to 80 °C, with a sweep frequency of 1 rad/s and shear stress of 5 s^−1^. The *G*′ and *G*″ values were recorded.

### 4.6. Water Holding Capacity (WHC)

The *WHC* was studied as follows: the gel was loaded into a 1.5 mL empty centrifuge tube and centrifuged at 10,000× *g* for 30 min with an ultracentrifuge. Then, the water was removed, and the mass of the remaining gel was recorded [37].

$$WHC(\%)=\frac{m_{2}-m_{0}}{m_{1}-m_{0}}\times 100\%$$

where *m*_0_ is the mass/g of the centrifuge tube, *m*_1_ is the total mass/g of the gel and centrifuge tube before centrifugation, and *m*_2_ is the total mass/g of the gel and centrifuge tube after centrifugation.

### 4.7. Scanning Electron Microscopy (SEM)

The surface morphology of the compound gels was visualized using a cold field emission SEM (EVOMA15, Carl Zeiss AG Co., Ltd., Oberkochen, Germany) operated at an accelerating voltage of 1 kV. Prior to detection, the gel samples, which were cut into thin slices with a diameter of 3 mm after freeze-drying, were fixed on copper stubs and sputtered with gold, and pictures were taken at 20× magnification.

### 4.8. Fourier Transform Infrared Spectroscopy (FTIR)

The chemical interactions between the TP functional groups and LGG were investigated using an FTIR spectrophotometer (Nicolet6700, Thermo Fisher Scientifific, MA, USA) as previously described [38], with some modifications. The FTIR studies of the gels were carried out by using the KBr pellet method. After freeze-drying, the gel samples were pressed with KBr and then scanned using an FTIR spectrometer in the scanning range of 400–4000 cm^−1^ and a resolution of 4 cm^−1^.

### 4.9. Statistical Analysis

All tests were conducted in a completely randomized design in independent triplicates to confirm the reproducibility of the results. The drawn figures in this article were processed using Origin Pro 8.0 (OriginLab Inc., Northampton, MA, USA). Duncan’s multiple range test determined significant differences, at the 95% confidential level (*p* < 0.05), using statistical SPSS software.

## Figures and Tables

**Table 1:** Color change of the TP-LGG compound gels with pH 3–9.

pH	L*	a*	b*	Δ*E*
3	12.28 ± 0.58 ^b^	2.36 ± 0.09 ^b^	10.55 ± 1.73 ^ab^	28.27 ± 0.08 ^b^
4	13.97 ± 0.90 ^ab^	2.35 ± 0.18 ^b^	11.89 ± 1.92 ^a^	28.56 ± 1.40 ^b^
5	13.95 ± 0.48 ^ab^	2.82 ± 0.84 ^ab^	11.32 ± 2.73 ^ab^	28.74 ± 0.26 ^b^
6	14.94 ± 0.58 ^a^	2.41 ± 0.49 ^b^	11.61 ± 1.77 ^a^	28.02 ± 1.41 ^b^
7	14.68 ± 0.94 ^a^	3.48 ± 0.21 ^a^	8.95 ± 0.32 ^ab^	28.59 ± 0.35 ^b^
8	13.20 ± 1.39 ^ab^	2.86 ± 0.29 ^ab^	7.33 ± 0.40 ^bc^	30.74 ± 0.87 ^a^
9	14.88 ± 0.88 ^a^	2.96 ± 0.06 ^ab^	5.33 ± 0.95 ^c^	31.01 ± 0.17 ^a^

The abc labeling method in statistics is a commonly used labeling method used to represent the significance and difference of statistical results. The difference between groups with the same letter is not significant, while the difference between groups with different letters is significant.

## Data Availability

Data are contained within the article.
